# Resolving Evolutionary Relationships in Closely Related Species with Whole-Genome Sequencing Data

**DOI:** 10.1093/sysbio/syv045

**Published:** 2015-07-17

**Authors:** Alexander Nater, Reto Burri, Takeshi Kawakami, Linnéa Smeds, Hans Ellegren

**Affiliations:** Department of Evolutionary Biology, Evolutionary Biology Centre, Uppsala University, Uppsala, Sweden

**Keywords:** Approximate Bayesian computation, demographic modeling, gene flow, gene tree, incomplete lineage sorting, introgression, phylogenomics, species tree

## Abstract

Using genetic data to resolve the evolutionary relationships of species is of major interest in evolutionary and systematic biology. However, reconstructing the sequence of speciation events, the so-called species tree, in closely related and potentially hybridizing species is very challenging. Processes such as incomplete lineage sorting and interspecific gene flow result in local gene genealogies that differ in their topology from the species tree, and analyses of few loci with a single sequence per species are likely to produce conflicting or even misleading results. To study these phenomena on a full phylogenomic scale, we use whole-genome sequence data from 200 individuals of four black-and-white flycatcher species with so far unresolved phylogenetic relationships to infer gene tree topologies and visualize genome-wide patterns of gene tree incongruence. Using phylogenetic analysis in nonoverlapping 10-kb windows, we show that gene tree topologies are extremely diverse and change on a very small physical scale. Moreover, we find strong evidence for gene flow among flycatcher species, with distinct patterns of reduced introgression on the Z chromosome. To resolve species relationships on the background of widespread gene tree incongruence, we used four complementary coalescent-based methods for species tree reconstruction, including complex modeling approaches that incorporate post-divergence gene flow among species. This allowed us to infer the most likely species tree with high confidence. Based on this finding, we show that regions of reduced effective population size, which have been suggested as particularly useful for species tree inference, can produce positively misleading species tree topologies. Our findings disclose the pitfalls of using loci potentially under selection as phylogenetic markers and highlight the potential of modeling approaches to disentangle species relationships in systems with large effective population sizes and post-divergence gene flow.

The application of genetic data to resolve the evolutionary relationships of species is of major interest in evolutionary and systematic biology. Traditionally, phylogenetic methods are applied to a single locus or a small number of loci and the branching patterns of the resulting phylogenetic trees are interpreted as though they reflect the chronological order of speciation events (Felsenstein 2004). This framework usually assumes a strictly bifurcating tree, which requires immediate and complete speciation events, and an instantaneous substitution process, which ignores many population-level effects, like gradual allele frequency changes (Felsenstein 1981; Golding and Felsenstein 1990). Such an approach might provide satisfactory results in distantly related taxa, but suffers from a number of issues when dealing with evolutionary relationships at shallow time depths. In such cases, a phylogenetic tree inferred from any given genomic locus (a ‘gene’ tree) might not unequivocally reflect the true order of speciation events (the ‘species’ tree), and inconsistent topologies are often obtained across the genome (Maddison 1997; Nichols 2001; Edwards 2009). This heterogeneity among gene trees also implies that the model underlying most phylogenetic approaches, which assumes that all sites in the alignment have evolved along the same tree, may be violated. As a result, the concatenation of multiple independent loci into a single ‘supermatrix’ can yield statistically inconsistent phylogenies and produce strongly misleading results when such an approach is applied to genome-wide data sets (Kubatko and Degnan 2007).

Genome-wide heterogeneity in observed gene tree topologies can be caused by multiple biological processes, but might also be a methodological artifact, because phylogenetic trees represent point estimates associated with uncertainty. Assuming that gene trees can be inferred correctly, comparison of paralogous sequences due to gene duplication and loss can cause gene tree discordance (Goodman et al. 1979), especially when trees are inferred over long evolutionary time scales. Within groups of closely related species, genome-wide variation in gene trees is caused mainly by two biological processes, namely incomplete lineage sorting (ILS) and interspecific gene flow.

ILS has been a major concern in the reconstruction of species trees in many taxonomic groups (Avise et al. 1983; Tajima 1983; Pamilo and Nei 1988; Maddison 1997). For instance, ILS has been studied intensively in great apes and humans, for which the first phylogenetic analysis using molecular data dates back more than 30 years (Ferris et al. 1981). Even in such clearly delimited species as humans, chimpanzees and gorillas, around 30% of loci throughout the genome support a topology that deviates from the well-supported species tree (Scally et al. 2012). ILS occurs when lineages fail to coalesce in the ancestral population of two species (Pamilo and Nei 1988; Maddison 1997; Degnan and Salter 2005). Therefore, the probability of ILS depends on both the effective population size ($N_{\text{e}}$) in the ancestral population of two species, which determines the rate of coalescence of lineages, and the time between two successive speciation events (internode length, Hudson 1990; Degnan and Salter 2005).

Although gene tree incongruences caused by ILS are still fully compatible with a strictly bifurcating species tree, gene flow among species requires a more complex representation of evolutionary histories, resembling reticulate networks rather than trees (Yu et al. 2011). If lineages from one species successfully introgress into another species and spread to high frequency, the genealogy of these lineages will support a clustering of species that exchanged genes after their initial split regardless of their evolutionary relationship. However, the rate of gene flow has been shown to vary along the genome, with repressed introgression in regions harboring genes involved in reproductive isolation or ecological specialization (Wu and Ting 2004; Via and West 2008; Nosil et al. 2009). Thus, in systems where hybridization occurs regularly, local gene trees will reflect the tendency of introgression at a specific locus. In contrast, if introgression is rare and selection for or against it is absent, the fate of an introgressed lineage is determined by chance alone, which might again produce a large variation in observed local gene trees.

A key parameter determining the distribution of gene trees along the genome is the recombination rate. Recombination affects gene tree topologies in three ways. First, recombination can physically join DNA segments with different genealogies on the same chromosome (Griffiths and Marjoram 1996; Zheng et al. 2014). Thus, in regions with a high population recombination rate gene tree topologies can change in quick succession along the genome. Second, regions with low recombination have been shown to have a lower $N_{\text{e}}$ as compared with high-recombination regions (Kaplan et al. 1989; Charlesworth et al. 1993; Charlesworth 2012). This is mainly due to the effects of selection, which affects large stretches of linked sequence in the absence of regular recombination. Due to enhanced lineage sorting, regions with low recombination rates and thus reduced $N_{\text{e}}$ will show less ILS and therefore more consistent topologies of local gene trees. Third, regions of low recombination are more likely to be resistant to introgression due to strong linkage to genetic incompatibilities or genes under divergent selection (Feder et al. 2012; Nachman and Payseur 2012). If specific loci within the genome of the introgressing species are detrimental on the genomic background of the other species, neighboring regions will only be able to introgress if recombination uncouples them from incompatibility loci before they are removed from the population by selection. In summary, regions of low recombination are expected to show gene trees consistent with the species tree in higher frequency than high-recombination regions, and these gene tree topologies will persist over longer physical distance. This renders such regions potentially useful to reconstruct species relationships in the presence of ILS and interspecific gene flow (Pease and Hahn 2013).

By modeling the distribution of gene trees under a given demographic model, coalescence-based modeling approaches can reconstruct species phylogenies by taking into account both ILS and interspecific gene flow. Additionally, given data from multiple independent loci and multiple individuals per species, such modeling approaches are able to harvest information about $N_{\text{e}}$ in both current and ancestral populations (Hudson 1990; Liu and Pearl 2007; Heled and Drummond 2010). However, due to computational constraints, multispecies coalescent methods have often been limited to relatively small data sets with only few loci (Rannala and Yang 2003; Liu and Pearl 2007; Liu 2008; Heled and Drummond 2010), and thus their usefulness for the analysis of whole-genome data is limited. Moreover, selection of a small number of loci might also strongly bias the outcome of the species tree inference (Maddison 1997; Nichols 2001; Degnan and Rosenberg 2009; Leache and Rannala 2011). Recent approaches have been optimized to utilize large numbers of independent loci. This considerably improves estimation of the species tree in closely related species where the variance in genealogies among loci is large (e.g., Liu et al. 2009; Liu et al. 2010; Bryant et al. 2012). These methodological advances therefore open the door to statistically sound phylogenomic analysis that avoids critical issues encountered when analyzing genome-wide data sets by concatenation and classical phylogenetic inference (Edwards et al. 2007; Kubatko and Degnan 2007; Rannala and Yang 2008; but see Gatesy and Springer 2014 for a counterargument).

A major limitation of many species-tree methods is their assumption of a model with strict isolation after species splits. It is unclear to what extent gene flow among lineages in the species tree can confound the true order of speciation events. Disentangling the effects of species split time and migration on genealogies is indeed challenging (Hey 2006). Even though maximum-likelihood modeling frameworks have been successfully applied to tackle this question, most of these approaches are restricted to a small number of populations or species, which limits their use to resolve phylogenetic questions (Hey and Nielsen 2004; Hey 2006). Furthermore, the calculation of the likelihood function is computationally expensive, and expanding such approaches to whole-genome data is therefore hindered by computational constraints. However, recent advances in approximate methods like approximate Bayesian computation (ABC) offer an elegant way around the problem of solving a likelihood function (Beaumont et al. 2002; Marjoram et al. 2003; Excoffier et al. 2013). This allows investigation of the influence of complex demographic processes on the reconstruction of species trees in closely related species, given the availability of both a large number of independent loci and a population sample from each species considered in the tree. However, despite recent advances in the generation of genome-wide sequencing data, such data sets remain rare and computational limitations have so far prevented a thorough investigation of the utility of coalescent-based approaches to resolve species relationships in closely related species.

Inferring phylogenetic relationships in birds has often been challenging due to rapid radiations and frequent hybridization (Grant and Grant 1992; Ericson et al. 2006). The Old World black-and-white flycatcher species complex (genus: *Ficedula*) represents such a case of closely related bird species with difficult-to-resolve evolutionary relationships. The four species of this complex—collared flycatcher (*F. albicollis*), pied flycatcher (*F. hypoleuca*), Atlas flycatcher (*F. speculigera*), and semicollared flycatcher (*F. semitorquata*)—warrant particular focus because of extensive phylogenetic, phylogeographic and ecological studies (e.g. Ellegren et al. 1996; Saetre et al. 1997; Qvarnstrom et al. 2000; Veen et al. 2001; Saether et al. 2007; Qvarnstrom et al. 2010; Saetre and Saether 2010). Black-and-white flycatchers are distributed in central and eastern Europe, the Middle East, and northwest Africa, and likely diverged from a common ancestor less than 2 Ma (Saetre et al. 2001). Like other species in Europe, they have likely experienced recurrent population expansions and contractions during Pleistocene glacial cycles, which resulted in recurrent episodes of isolation in refugia followed by hybridization upon secondary contact (Saetre et al. 2001; Borge et al. 2005; Backström et al. 2013; Nadachowska-Brzyska et al. 2013). Therefore, their recent divergence and repeated occurrence of hybridization make evolutionary relationships of black-and-white flycatchers difficult to resolve despite extensive studies using a wide variety of nonmolecular and molecular markers (Lundberg and Alatalo 1992; Saetre et al. 2001; Hogner et al. 2012; Smeds et al. 2015).

Here we used whole-genome sequence data of 200 individuals covering all four species of the black-and-white flycatcher species complex, as well as two outgroup species, to tackle the long-lasting problem of unravelling evolutionary relationships in closely related and rapidly radiating species. We aimed to quantify the genome-wide extent of gene tree discordance caused by ILS and interspecific gene flow in relation to chromosome location, within-species diversity, and recombination rate. Moreover, we apply a range of coalescent-based modeling approaches to find the species tree which best explains the observed distribution of local gene trees. By applying population genomic data, we aimed for two key improvements over earlier smaller scale studies. First, we avoid biases in *a priori* selection of specific loci and additionally obtain information about genome-wide variation in genealogies, which aids in selecting suitable loci in other species with less extensive data. Second, by employing sequence data from multiple individuals per species, we identify genomic regions of accelerated lineage sorting in which signals of speciation events should be pronounced. Moreover, population sampling allows estimation of species-specific $N_{\text{e}}$ and gene flow rates, which aids in disentangling species split patterns from overlaying signals of introgression.

Our findings highlight the difficulties and pitfalls encountered when dealing with phylogenetic questions in closely related and hybridizing species. Owing to the availability of extensive population genomic resources, black-and-white flycatchers can serve as a model system to guide the selection of appropriate genetic markers and analysis methods for systems with similarly challenging evolutionary relationships but lacking comparable data.

## Materials and Methods

### Sampling

The data set included 79 collared flycatchers (*F. albicollis*, abbreviated as C) from four European populations (Italy, Hungary, Czech Republic, and the Baltic Sea island Öland), 79 pied flycatchers (*F. hypoleuca*, [P]) from four European populations (Spain, Czech Republic, Swedish mainland, and Öland), 20 Atlas flycatchers (*F. speculigera*, [A]) from Northwest Africa, and 20 semicollared flycatcher (*F. semitorquata*, [S]) from Bulgaria. Additionally, we included a single individual each of red-breasted flycatcher (*F. parva; *Natural History Museum of Stockholm, accession NRM996601) and snowy-browed flycatcher (*F. hyperythra; *Zoological Museum of the University of Copenhagen, accession 148317) as outgroup species. Samples consisted of either blood or tissue. Further details about sampling localities and sample storage are provided in Table S1 (available as Supplementary Material on Dryad at http://dx.doi.org/10.5061/dryad.b6gj8).

### Sequence Data and General Filtering Strategy

We extracted DNA from the blood and tissue samples using Qiagen's Blood and Tissue Kit following the manufacturer's instructions. We performed whole-genome sequencing using paired-end libraries with 450-bp insert size on an Illumina HiSeq 2000 instrument with 100-bp read length. We sequenced the 200 individuals at a mean depth of 14.7× per individual (min 5.0×, max 26.7×, Table 1, Table S1) and mapped reads against the collared flycatcher assembly version FicAlb1.5 (Kawakami et al. 2014b) using BWA 0.7.4 (Li and Durbin 2009). We then performed variant calling on a per-population/species level using GATK 2.8-1 (McKenna et al. 2010), following the GATK Best Practices workflow for DNAseq data. This included a variant quality recalibration and filtering step using high-quality genotype data from a 50k SNP chip (Kawakami et al. 2014a) to obtain high-quality single-nucleotide polymorphisms (SNPs). To further reduce erroneous genotype calls, we applied a common filter to all sequences used in subsequent analyses. This included a minimum root mean square of mapping quality of 20 per site, a minimum read coverage of 5× per individual and site, and a minimum number of 10 individuals with callable genotype per population/species. Sites not conforming to these filter requirements were coded as missing. To remove potentially collapsed regions in the reference genome assembly, we additionally excluded all sites with a sequencing coverage larger than five standard deviations from the mean coverage across all individuals or with exclusively heterozygous genotypes in any of the populations/species. Furthermore, we applied a repeat mask to the collared flycatcher reference sequence as described in Ellegren et al. 2012.

**Table 1.** Sampling scheme and summary statistics for the four flycatcher

Species/Population	$N_{\text{Samples}}$^a^	Seq. coverage^b^	$N_{\text{SNPs}}$^c^	π^d^	$\theta_{\text{W}}$^e^
Collared	79	14.4×[5.9−23.8]	15,201,728	0.0041	0.0045
Italy	20	13.9×[7.6−21.4]	14,672,912	0.0043	0.0045
Hungary	20	13.4×[9.3−23.8]	14,250,553	0.0042	0.0044
Czech Republic	20	15.0×[5.9−20.7]	14,181,354	0.0042	0.0044
Öland	19	15.5×[6.7−23.8]	12,317,929	0.0040	0.0038
Pied	79	14.1×[6.3−26.7]	12,046,196	0.0032	0.0036
Spain	20	13.0×[6.3−23.1]	9,963,741	0.0032	0.0032
Sweden	20	16.0×[10.2−26.7]	10,404,068	0.0032	0.0033
Czech Republic	20	14.4×[10.5−23.0]	9,708,126	0.0031	0.0031
Öland	19	12.9×[7.6−20.9]	10,271,020	0.0032	0.0033
Atlas	20	20.9×[16.2−26.2]	7,789,570	0.0031	0.0029
Semicollared	20	12.3×[5.0−17.7]	8,561,750	0.0032	0.0034

^a^Number of sampled individuals; ^b^mean and range of sequencing coverage; ^c^number of single-nucleotide polymorphisms within species/populations after all filtering steps; ^d^nucleotide diversity per base pair; ^e^Watterson's θ per base pair.

### Polarization

We used the two outgroup genomes to determine the ancestral state for each position in the reference genome using a parsimony-based approach. If both outgroup individuals were monomorphic for the same allele, this allele was considered ancestral. If data were missing, one of the outgroups was polymorphic, or the two outgroups were fixed for different alleles, the ancestral state was considered undetermined and the position coded as missing data in all individuals.

### Allele Sharing and Principal Component Analysis

We used a custom-made Perl script to count the occurrences of shared derived variation in any pairwise comparison of the four focal species using SNP filtering and polarization as described above. Venn diagrams were plotted using the R package VennDiagram (Chen and Boutros 2011).

To obtain a genome-wide picture of the partitioning of genetic diversity among species, we performed a principal component analysis (PCA) using the “prcomp” function in R v3.0.1 (R Development Core Team 2010). For this, we generated a table of diploid genotype calls for a total of 10 million SNPs with complete genotypes in all 198 individuals of the four focal species. We randomly sampled SNPs along the genome, but excluding the Z chromosome, mtDNA genome, and regions with elevated differentiation (differentiation islands) as defined by Ellegren et al. 2012.

### Window-based Gene Tree Discordance

We assessed the genome-wide distribution of gene tree topologies with a stepping window approach, moving 10-kb windows in steps of 50 kb to minimize linkage between subsequent windows. For each window, we first inferred haplotypes of all 198 individuals of the focal species using fastPHASE v1.4.0 (Scheet and Stephens 2006). To minimize phasing errors, we coded sites with less than 80% posterior phasing support as missing data and randomly selected one haplotype per individual. Due to unequal sample sizes among species, we subsampled pied and collared flycatchers by selecting for each window and each of the four populations per species the five individuals with the highest sequencing coverage. Employing the alignment of haploid sequences, we then inferred the maximum-likelihood gene tree as well as 200 bootstrap replicates using the rapid bootstrapping algorithm with the GTRGAMMA model in RAxML v8.0.20 (Stamatakis 2014). We rooted the resulting trees with the *F. hyperythra* outgroup using Newick Utilities v1.6 (Junier and Zdobnov 2010). We processed the rooted trees using a custom-made Perl script to calculate the genealogical sorting index (gsi, Cummings et al. 2008) for each of the four species, as well as the depth of the most recent common ancestor of all four species relative to the root depth. The gsi is a standardized measurement of the degree of monophyly for a group of lineages on a given gene tree topology, with a value of one indicating that the lineages form a monophyletic group, whereas a value of zero indicates a random distribution of lineages across the entire tree.

Because lack of species monophyly is common in this study system, we performed 200 rounds of subsampling for each gene tree by randomly selecting one leaf node per species, including the *F. parva* and *F. hyperythra* outgroups. We extracted the same leaf nodes from the 200 bootstrap trees and used these replicates to annotate bootstrap support values on the original six-taxon tree using Newick Utilities. We then processed each subsampled tree with a custom-made Perl script using the BioPerl Tree module (Stajich et al. 2002) in order to determine the frequencies of the rooted and unrooted tree topologies over the 200 iterations for each window. Based on the frequencies (p) of gene tree topologies in 10-kb windows, we calculated a topology diversity index (tdi) as $\frac{1-\sum_{i=1}^{I}p_{i}^{2}}{1-\sum_{i=1}^{I}{\left(\frac{1}{I}\right)}^{2}}$, with I being the number of possible rooted tree topologies. A value of one therefore indicates the occurrence of all possible topologies at equal frequencies, whereas a value of zero means that only a single topology is found in a window. We calculated the tdi for both single 10-kb windows, as well as with frequencies averaged over 500-kb sliding windows to assess persistence of topologies over longer physical distance. We used R to plot all window-based statistics of gene tree distributions.

In order to test whether a quantitative analysis of the genome-wide distribution of gene tree topologies can be used for species tree inference, we summed the frequencies of the 15 possible rooted and three unrooted gene tree topologies over the entire genome, omitting every other window in order to reduce linkage effects. We also assessed if a particular tree topology was enriched in low-recombining or highly differentiated regions of the genome as suggested before (Corl and Ellegren 2013; Pease and Hahn 2013; Cruickshank and Hahn 2014). For this, we partitioned the window-based tree topologies into differentiation islands as defined in Ellegren et al. 2012, the lower 10% quantile of recombination rates (< 0.71 cM/Mb, Kawakami et al. 2014b), and the Z chromosome. Additionally, we applied a supermatrix approach to obtain genome-wide phylogenetic trees by concatenating individual haplotypes from 10-kb windows in each of the four data partitions. Due to the large number of loci in the complete data set, we subsampled by concatenating only every 10th window. We then used the GTRCAT model in RAxML to infer the maximum-likelihood tree as well as 200 rapid bootstrap replicates.

### Model-based Species Tree Reconstruction

We applied a series of modeling approaches suitable for whole-genome data in order to test the power to resolve the species tree in a group of closely related species. All modeling approaches are based on the multispecies coalescent, but employ different population models and marker types for inference. With the exception of the MP-EST analysis, for which we used the same data partitions as in the window-based analyses, we applied stringent filters to select suitable loci for the model-based species tree inference. Because these models assume neutrality of the employed genetic markers, we only considered loci in the genome that were at least 20 kb away from any exonic region as determined by the Ensembl gene annotation of the collared flycatcher reference genome version FicAlb1.4. We also excluded loci on the Z chromosome and the mitochondrial genome, as these are expected to have reduced $N_{\text{e}}$ as compared with autosomal regions. Furthermore, we did not consider any loci located within differentiation islands as defined by Ellegren et al. 2012, as these show patterns of diversity and differentiation clearly distinct from the genomic background, which might be the results of selective sweeps or background selection.

### MP-EST

As a first approach, we applied MP-EST v1.4 (Liu et al. 2010) to the set of 18,649 gene trees from 10-kb windows obtained in the previous analysis. Given a set of gene trees, MP-EST optimizes a pseudo-likelihood function of the species tree using the multispecies coalescent model. Thus, MP-EST accounts for ILS as a source of gene tree/species tree discordance, but does not model interspecific gene flow. MP-EST is computationally highly efficient, which allowed us to analyze the genome-wide distribution of gene tree topologies in a species tree framework. We performed ten independent runs each on the four different partitions (all regions, low-recombination regions, differentiation islands, and Z chromosome), retaining the tree with the highest pseudo-likelihood for each partition. Due to the large number of available independent loci and the short run times of the MP-EST method, we additionally explored the precision of inferring a given species tree relative to the number of loci used. For this, we subsampled from the full set of trees in each of the four partitions 1000 times without replacement between 10 and 1000 gene trees. We then analyzed each of the resulting 10,000 data sets with MP-EST in the same way as for the complete data sets.

### SNAPP

We used SNAPP v1.1.1 (Bryant et al. 2012), which assumes a strict isolation model with constant population sizes to estimate the species tree using a large number of independent genetic markers. SNAPP calculates the probability of the data given a species tree, bypassing the need to integrate over all possible genealogies. As a major advantage, SNAPP performs tree search and therefore does not require *a priori* definition of a set of species tree topologies to test. Additionally, SNAPP provides estimates of theta for all current and ancestral species; theta equals $4N_{\text{e}}\mu$, with μ being the mutation rate per site per generation.

Due to severe runtime and memory constraints of this method, we used a relatively small data set of 16,000 SNPs randomly sampled from the genome. We collected the allelic states for each SNP in a random subset of 12 individuals per species, and from each individual we randomly sampled one of the two chromosomes. SNPs with less than 12 covered individuals per species were not considered. We coded allelic states as either ancestral or derived as described above. For the priors of the model parameters, we chose wide and uninformative distributions. The forward and backward mutation rates were sampled from uniform distributions between zero and 100, and the rate parameters were sampled from a gamma distribution with the alpha and beta parameters fixed at 10 and 100, respectively. The lambda parameter of the Yule prior for the species tree was uniformly distributed in the range of zero to one. We performed 10 independent runs, each with 1,000,000 iterations of the Markov chain Monte Carlo (MCMC) algorithm, discarding the first 200,000 iterations of each run as burn-in. We then assessed convergence of the MCMC chain using Tracer v1.5 (Rambaut and Drummond 2007) and plotted the distribution of species trees in the posterior sample using DensiTree v2.1.11 (Bouckaert 2010).

### Fastsimcoal2

To make more efficient use of our genome-wide data set, we used an approximate method to fit demographic models to the observed two-dimensional site frequency spectra (2D-SFS) as implemented in fastsimcoal v2.5.2 (Excoffier et al. 2013). This approach approximates the probability of observing a certain site frequency configuration given a demographic model by simulating a large number of genealogies for each model/parameter setting. The length of the branches leading to the number of tips matching the site frequency configuration relative to the total length of the genealogy is then averaged over all simulated genealogies. Assuming independence of SNPs, a composite likelihood can be calculated by multiplying the approximate likelihoods for each cell in the SFS. This composite likelihood is then maximized for the whole 2D-SFS (or six 2D-SFS in our case—one for each pairwise comparison of four species) to obtain maximum-likelihood estimates of the model parameters. Because we also scored the total number of monomorphic sites (the 0/0 and 1/1 boxes in the 2D-SFS) and an estimate of the mutation rate was available ($2.5\times 10^{\text{-9}}$ per site per generation, Nadachowska-Brzyska et al. 2013), this method is also able to provide absolute estimates of all the parameters in the model. Due to its approximate nature, complex demographic models can be assessed, including gene flow among species, which enabled us to test for the influence of gene flow in species tree reconstruction.

Composite likelihood methods like fastsimcoal2 assume independence of the sites in the SFS. We therefore assessed a subset of sites distributed randomly across the genome. For this, we first divided the genome into 100-bp windows, filtered all windows with more than 20% repeat-masked sites, and then randomly sampled 100,000 windows without replacement (from a total of 3,282,941 windows). In each 100-bp window, we subsampled each position to 24 random chromosomes per species and counted the number of derived alleles in each species for each pairwise comparison. Positions that did not have enough data for one or more of the four species were excluded in all species.

Because fastsimcoal2 does not allow for exhaustive tree search, we restricted our model testing procedure to the four most frequently observed gene tree topologies as determined in the genome-wide analysis of gene tree discordance. The search ranges for the model parameters are listed in Table S2. We optimized the composite likelihood for each model in 100 independent runs, each using 200,000 simulations for every cycle in the expectation conditional maximization (ECM) algorithm, a minimum of 10 and a maximum of 40 ECM cycles, and a stop criterion of 0.001. We then compared the run with the highest likelihood among the four models to obtain the Akaike's weight of evidence for the model with the highest likelihood (Johnson and Omland 2004). To obtain confidence intervals for the parameter estimates of the best-fitting model, we applied a re-subsampling procedure of our full set of genome-wide 100-bp windows. We repeated the subsampling of 100,000 windows a total of 50 times and obtained maximum-likelihood estimates for each replicate as described above, but with only 30 independent runs per replicate to reduce computation time.

### ABC

As a fourth model-based approach to species tree reconstruction, we applied an ABC framework (Beaumont et al. 2002). Following a recent simulation study (Robinson et al. 2014), we gathered a total of 2962 independent sequence loci of 2 kb each from 12 individuals per species. We randomly sampled loci from the genome, with a minimum distance of 50 kb between loci to minimize the effects of linkage. We then calculated the mean and the variance over all loci of the following summary statistics: number of segregating sites, proportions of shared and fixed polymorphism, average sequence divergence ($d_{\text{XY}}$), and $\Phi_{\text{ST}}$ (Excoffier et al. 1992). We calculated the statistics for each of the six pairwise species comparisons, thus resulting in a total of 60 summary statistics used in the ABC analysis.

As in the fastsimcoal2 analysis, we restricted the model testing to the four most frequent gene tree topologies. The prior distributions for the model parameters are listed in Table S3. We simulated $2\times 10^{\text{6}}$ data sets for each model using the coalescent simulator ms (Hudson 2002). From the simulated data, we calculated summary statistics using a custom-made C++ program. We then used the R package “abc” v1.8 (Csillery et al. 2012) to perform a multinomial logistic regression on the simulated and observed summary statistics, using a tolerance level of 0.1% (8000 simulations closest to the observed data). For parameter estimation, we performed another $8\times 10^{6}$ simulations for the best-fitting model. We used 20,000 random simulations to define the first 10 orthogonal components of the summary statistics that maximize the covariance matrix between summary statistics and model parameters, using a partial least-squares (PLS) regression approach (Boulesteix and Strimmer 2007) as implemented in the “pls” R package (Mevik and Wehrens 2007). We defined the optimal number of PLS components based on the drop in the root mean squared error for each parameter with the inclusion of additional PLS components (Wegmann et al. 2009). We then transformed the simulated summary statistics with the loadings of the first 10 PLS components and performed a ABC-GLM post-sampling regression (Leuenberger and Wegmann 2010) on the 8000 simulations with the smallest Euclidean distance to the PLS components of the observed summary statistics.

We assessed the power of our model selection approach to differentiate between the four tested demographic models by a cross-validation procedure. For each model, we randomly selected 200 simulated data sets and performed the same model selection procedure as with the real observed data. For each pseudo-observed data set, we scored the model with the highest posterior probability as well as the mean posterior probability of the true model (Fig. S5).

## Results

### Allele Sharing and PCA

**Figure 1.**
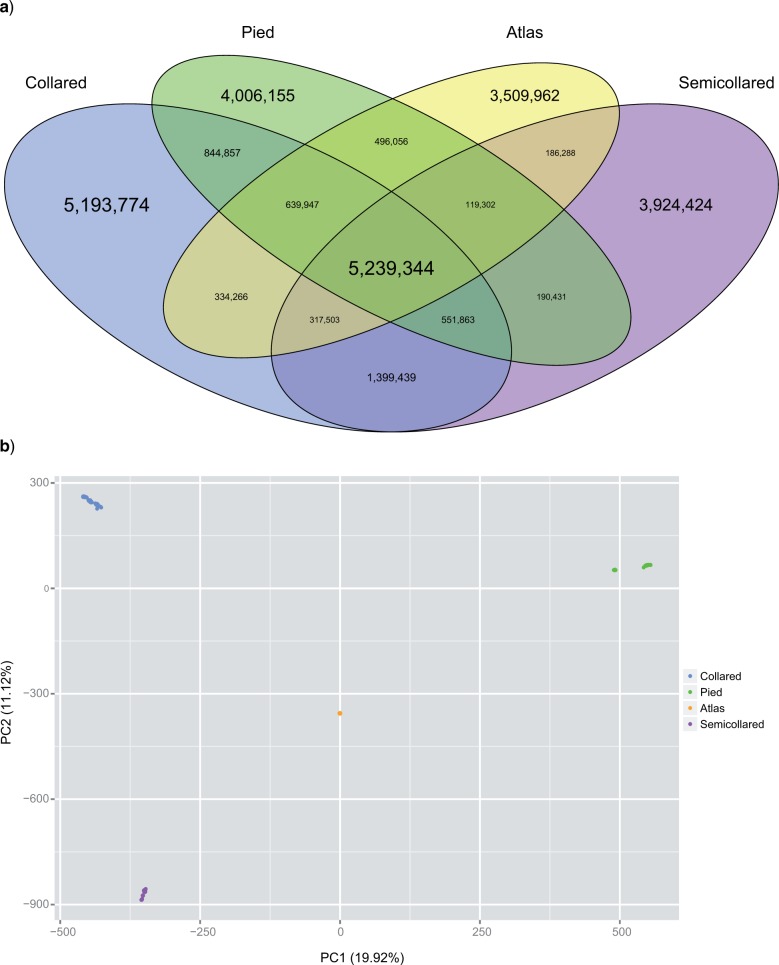
a) Sharing of derived variation among the four flycatcher species. The number in each field depicts the number of SNPs with a shared derived allele (fixed or polymorphic) present in the corresponding species. Only SNPs where the ancestral state could be determined are shown (N= 26,953,611). The font size is approximately proportional to the number of SNPs in each field. b) PCA plot of the first two principal components of 10 million SNPs.

In a first step, we assessed the degree of pairwise allele sharing between the four flycatcher species, which is indicative of the prevalence of ILS and/or interspecific gene flow. We used ancestral state information from two outgroup species to polarize SNPs and specifically look at shared and private occurrence of derived alleles (Fig. 1a). Out of a total of ∼27 million SNPs polymorphic over all four species, ∼5.2 million derived variants are shared among all four species, highlighting the close evolutionary relationship of black-and-white flycatchers. Between 3.5 and 5.2 million SNPs were private to a single species, with collared flycatcher showing the highest number of SNPs with private derived variation. In pairwise comparisons, most derived variation was shared between collared flycatcher and semicollared flycatcher (∼1.4 million SNPs), followed by collared flycatcher and pied flycatcher (∼0.85 million SNPs).

A PCA of 10 million genome-wide SNPs revealed a strong clustering of individuals by species with no overlap (Fig. 1b). The first principal component (PC), explaining ∼20% of the total variance, separated pied, Atlas, as well as collared and semicollared flycatchers into three distinct groups. PC1 also revealed strong population structure within pied flycatchers, with samples from the Spanish population being genetically distinct from the other samples. PC2 (∼11% of the variance) separated semicollared from Atlas flycatchers, but kept collared and pied flycatchers together. Atlas flycatchers were located centrally in the plot of the first two components, approximately equidistant to the other three species.

### Genome-wide Gene Tree Incongruence

**Figure 2.**
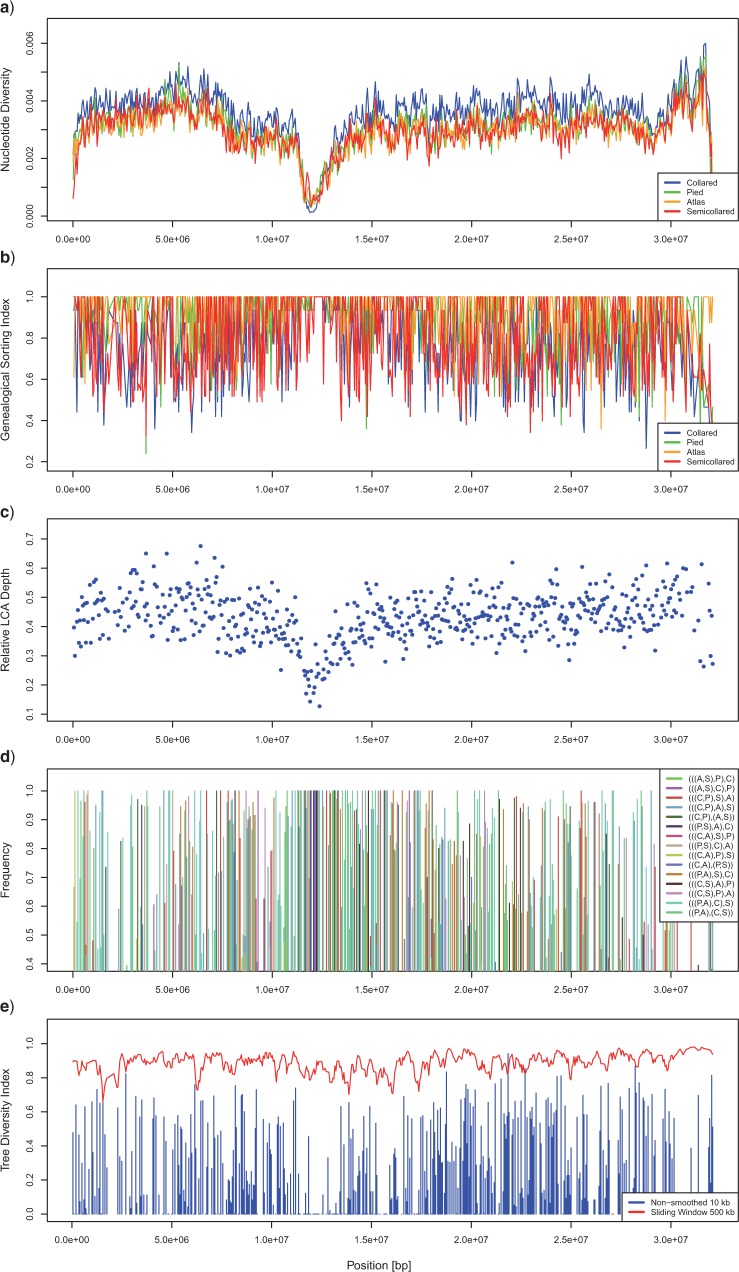
Distribution of nucleotide diversity (π, panel A), genealogical sorting index (gsi, panel B), depth of the lowest common ancestor (LCA) of all four flycatcher species relative to the root depth (panel C), frequencies of all 15 possible rooted tree topologies (panel D), and topology diversity index (tdi, panel E) along an example chromosome (Chromosome 8). For panels D and E, only windows with gene trees exhibiting an average bootstrap support of 50 or higher for the two internal branches defining species relationships are shown.

Assessment of the genome-wide distribution of gene tree topologies revealed widespread gene tree incongruence. The four species did not form monophyletic groups for most genomic regions, as evidenced by genealogical sorting indices (gsi) below one (Fig. 2b). For most genomic regions, none of the 15 rooted gene tree topologies appeared consistently at high frequencies, as shown by tree diversity indices well above zero (Fig. 2e, Fig. S6). Rather, the genome-wide gene tree distribution was characterized by narrow frequency spikes of quickly changing topologies, indicating widespread gene tree discordance in the four species (Fig. 2d, Fig. S6). This result cannot be explained by unreliable placement of the root node, as the three possible unrooted tree configurations showed similar diversity along the genome (Fig. S7).

Despite widespread lack of species monophyly, both gsi and tdi identified regions in the genome that displayed strongly increased rates of lineage sorting (Fig. 2b, Fig. S6). These regions were accompanied by reduced within-species diversity (nucleotide diversity, π, Fig. 2a) and largely corresponded to previously identified differentiation islands (Ellegren et al. 2012). Differentiation islands are strongly associated with genomic regions of low recombination (Kawakami et al. 2014a), and are likely the result of locally reduced $N_{\text{e}}$ due to the effects of selection at linked sites. Within differentiation islands, windows with gsi values of one for all four species were common, indicating accelerated lineage sorting due to strongly reduced current effective population sizes. Although the reduction in $N_{\text{e}}$ within differentiation islands was strong enough to promote species monophyly, we still observed switching of tree topologies in quick succession within these regions. This is highlighted by the tdi calculated over sliding 500-kb windows, which stayed close to one even in regions where multiple successive 10-kb windows showed a tdi of zero. A similar, but chromosome-wide, pattern of accelerated lineage sorting was visible on the Z chromosome, in line with the reduced within-species diversity and increased differentiation on this chromosome (Backström et al. 2010; Ellegren et al. 2012).

**Figure 3.**
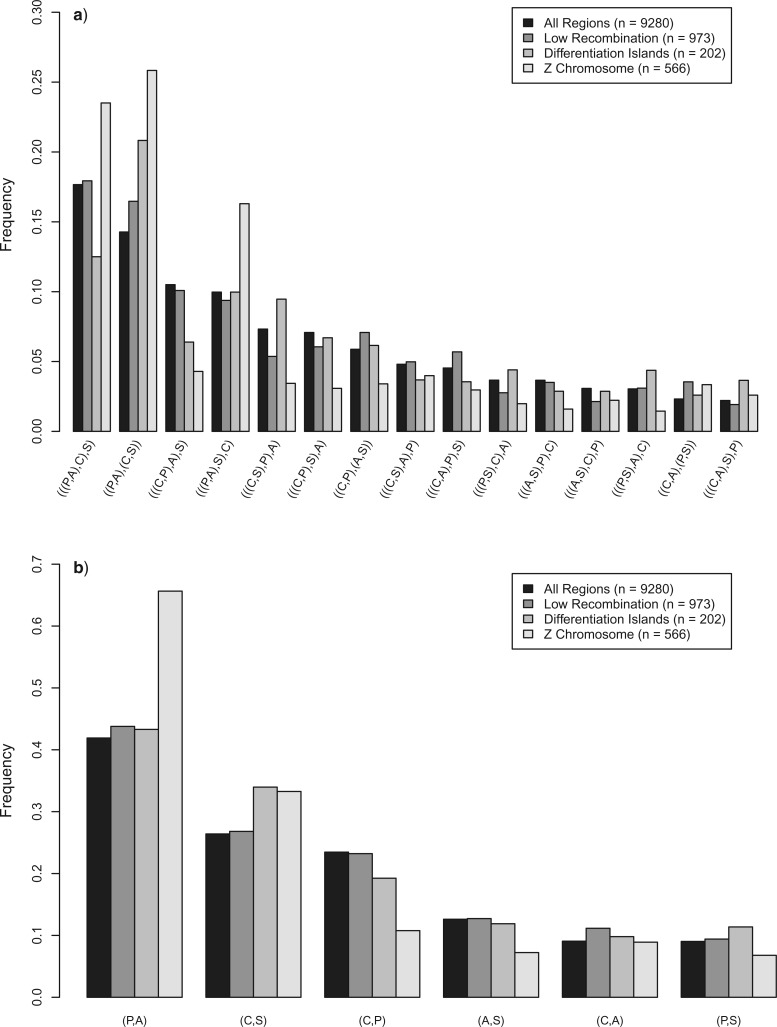
Genome-wide proportions of gene tree topologies for four different data partitions. The topologies are ranked by their frequency in the whole-genome data set. a) Frequency distribution of rooted gene tree topologies. b) Frequency distribution of species pairings in rooted gene tree topologies.

A quantitative assessment of the distribution of gene tree topologies along the genome revealed that an asymmetric gene tree topology (((P,A),C),S) was most common (17.7%, Fig. 3a). This contrasted with an expected frequency of ∼5.5% in a four-taxon tree with internal branch lengths of zero (Degnan and Rosenberg 2006). The second-ranked, symmetric topology ((P,A),(C,S)) occurred with a frequency of 14.3% versus the expected ∼11%. In combination, the distribution of gene tree topologies strongly supported a species tree with an internal pairing of pied flycatcher and Atlas flycatcher (41.9% of all gene trees, Fig. 3b). However, the third-ranked topology (((C,P),A),S) was still nearly 2-fold overrepresented (10.5% vs. ∼5.5%), even though such a gene tree topology is discordant to a species tree with an internal pied–Atlas pairing. In total, 23.5% of all gene trees supported an internal collared–pied pairing (Fig. 3b). In agreement with the observed frequencies of gene trees, the phylogenetic tree inferred from the genome-wide concatenated data set resulted in a highly supported asymmetric (((P,A),C),S) topology (Fig. S2).

When restricting the quantitative analysis to specific partitions of the genome, the frequency distribution of gene trees changed considerably as compared with the whole-genome analysis (Fig. 3a). For low-recombination regions (recombination rate <0.71 cM/Mb), the ranking order of the four most frequent topologies stayed the same, but the symmetrical ((P,A),(C,S)) topology occurred almost as frequently as the top-ranking asymmetrical (((P,A),C),S) tree (16.5% vs. 17.9%). However, for differentiation islands and the Z chromosome, the order of the first two topologies switched, with the symmetrical tree being the most frequent (20.8% vs. 12.5% and 25.8% vs. 23.5%, respectively). For the Z chromosome, an increase in all topologies with an internal pied–Atlas pairing was evident (65.6% vs. 41.9% for all genomic regions, Fig. 3b), with a corresponding underrepresentation of trees with an internal collared–pied pairing (10.8% vs. 23.5%). Such a pattern of increased occurrence of a certain gene tree topology in regions with reduced $N_{\text{e}}$ and therefore ILS (which applies to all three mentioned regions) lends strong support that this particular configuration reflects the true species relationships. This notion is further supported by the phylogenetic trees obtained from concatenated 10-kb loci, which resulted in the same symmetrical ((P,A),(C,S)) topology with 100% bootstrap support for all three restricted data partitions (Fig. S2). However, in the following paragraphs, we show that such a conclusion would be misleading in the case of black-and-white flycatchers.

### Model-based Species Tree Reconstruction

**Figure 4.**
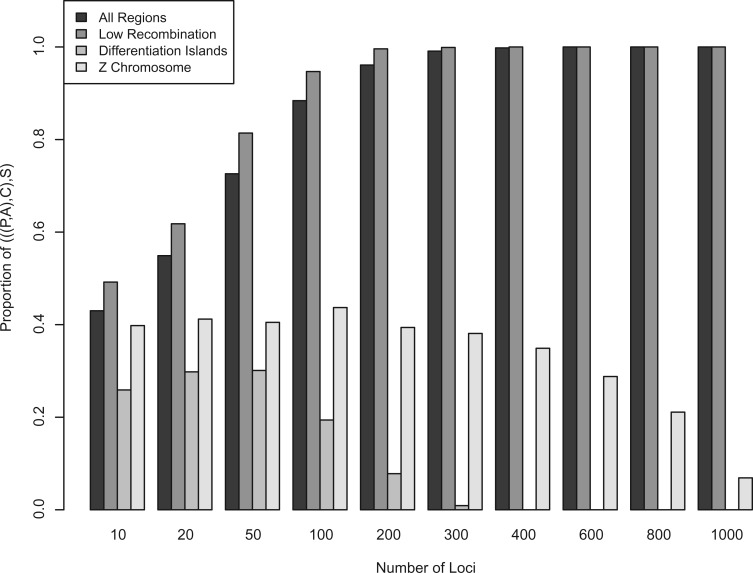
Re-subsampling of 10-kb loci for species tree inference wit MP-EST. For each number of loci, gene trees were re-subsampled without replacement 1,000 times from the complete set of gene trees of four data partitions. The proportion of resulting species trees matching the topology obtained with the complete data set (((P,A),C),S) is shown for 10 different numbers of loci. For the differentiation islands and the Z chromosome, species trees converged to the alternative ((P,A),(C,S)) topology with increasing number of loci.

Analyzing the genome-wide collection of gene trees from 10-kb windows in a species tree framework implemented in MP-EST confirmed the results obtained from the frequency distributions. Although we obtained an asymmetrical (((P,A),C),S) species tree with the genome-wide and the low-recombination regions data sets, the topology switched to a symmetrical ((P,A),(C,S)) tree for the differentiation islands and the Z chromosome (Fig. S3). Subsampling smaller numbers of independent loci revealed that the corresponding topologies obtained with the complete data sets were consistently (>95% of replicates) obtained with 200 loci sampled genome-wide and within differentiation islands (Fig. 4). The low-recombination regions had higher power to infer the same species tree, reaching a 95% confidence level already at 100 loci. Interestingly, the Z chromosome converged slower to the topology obtained with the complete set of Z-chromosomal gene trees, requiring over 1000 loci to reach the 95% confidence level.

**Figure 5.**
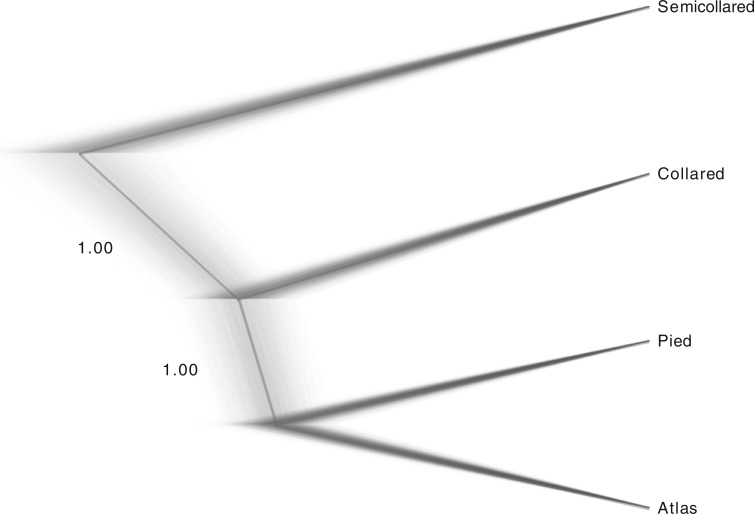
Distribution of species tree topologies from the posterior sample of the SNAPP analysis (thin gray lines). The thick gray line shows the consensus tree with averaged branch lengths. Clade support is provided as Bayesian posterior probabilities next to the two internal branches.

Species tree reconstruction based on 16,000 genome-wide SNPs with SNAPP, a modeling approach assuming strict isolation after species splits, resulted in a well-resolved topology. After removing the burn-in, the MCMC sample only contained species trees with an asymmetric (((P,A),C),S) topology (Fig. 5). Theta estimates from the SNAPP analysis revealed similar sizes for modern and ancestral populations, except for collared flycatchers, which showed a markedly larger current effective population size (Table S4). Three additional runs each with a new random sample of genome-wide SNPs resulted all in the same (((P,A),C),S) topology and highly similar estimates of theta (Table S4).

To assess the impact of interspecific gene flow on species tree inference, we applied two more complex modeling approaches. First, we used fastsimcoal2 to fit demographic models representing the four most frequent gene tree topologies to pairwise 2D-SFS, which provided strong support for the same species tree topology as inferred by SNAPP (Table 2). We obtained low maximum-likelihood estimates for all migration rates among species, indicating that gene flow among species has not been strong enough to influence the coalescent patterns in the data in a way that would provide spurious results in models without gene flow. In accordance with this notion, we obtained similar patterns of ${\text{N}}_{e}$ estimates as in the SNAPP analysis, with collared flycatchers showing considerably larger $N_{\text{e}}$ than the other species (collared: ∼450,000; pied: ∼300,000; Atlas/semicollared: ∼250,000; Table 3). Fastsimcoal2 inferred very short internode lengths of just $∼0.17N_{\text{e}}$ generations for the pied–Atlas branch and $∼0.30N_{\text{e}}$ generations for the pied–Atlas–collared branch.

**Table 2.** Model testing of the four most frequent species tree topologies with fastsimcoal2 and ABC

Model	Freq^a^	ABC PP^b^	ML^c^	ΔAIC^d^	${\text{w}}_{i}$^e^
(((P,A),C),S)	0.177	0.75	−4,052,489	0	≫0.999
((P,A),(C,S))	0.143	0.22	−4,053,548	2120	0.000
(((C,P),A),S)	0.105	0.03	−4,052,706	434	$5.62\times 10^{-95}$
(((P,A),S),C)	0.099	0.00	−4,053,509	2041	0.000

^a^Frequency of the gene tree topology in the whole-genome data set; ^b^model posterior probability from the ABC analysis; ^c^natural logarithm of maximum likelihood of optimized model from the fastsimcoal2 analysis; ^d^difference in Akaike information criterion relative to the best-fitting model; ^e^Akaike's weight of evidence based on maximum-likelihood estimate.

**Table 3.** Parameter estimation from fastsimcoal2 and ABC for the inferred species tree model (((P,A),C),S)

Parameter^a^	Prior^b^	Mode^c^	90%-HPD^d^	MLE^e^	90%-CI^f^
${\text{N}}_{\text{NOW}}$C	log-uniform [30,000–1,000,000]	365,831	178,908–701,730	449,385	390,266–463,979
${\text{N}}_{\text{NOW}}$P	log-uniform [30,000–1,000,000]	185,836	98,230–352,184	304,853	263,951–318,282
${\text{N}}_{\text{NOW}}$A	log-uniform [30,000–1,000,000]	138,299	72,224–298,360	244,520	205,829–249,540
${\text{N}}_{\text{NOW}}$S	log-uniform [30,000–1,000,000]	108,211	50,073–285,746	238,722	203,544–242,792
${\text{N}}_{\text{ANC}}$PA	log-uniform [100,000–3,000,000]	1,205,841	224,481–2,831,262	256,334	215,572–338,725
${\text{N}}_{\text{ANC}}$PAC	log-uniform [100,000–3,000,000]	648,500	155,364–2,457,254	1,067,020	821,369–1,091,696
${\text{N}}_{\text{ANC}}$PACS	log-uniform [30,000–1,000,000]	268,974	93,753–696,899	304,660	261,469–315,043
${\text{T}}_{\text{SPLIT}}$P/A	uniform [144,000–720,000]	392,380	185,205–645,083	561,326	482,324–581,711
${\text{T}}_{\text{SPLIT}}$PA/C	uniform [360,000–1,080,000]	577,909	386,293–888,026	638,933	557,150–660,946
${\text{T}}_{\text{SPLIT}}$PAC/S	uniform [720,000–1,800,000]	1,336,990	815,630–1,731,924	1,218,623	1,030,427–1,215,023
${\text{Nm}}_{\text{CP}}$	log-uniform [0.001–10]	0.025	0.002–0.551	0.538	0.461–0.541
${\text{Nm}}_{\text{PC}}$	log-uniform [0.001–10]	0.008	0.001–0.128	0.124	0.126–0.170
${\text{Nm}}_{\text{PA}}$	log-uniform [0.001–10]	0.006	0.001–0.111	0.047	0.011–0.065
${\text{Nm}}_{\text{AP}}$	log-uniform [0.001–10]	0.016	0.002–0.369	0.168	0.149–0.197
${\text{Nm}}_{\text{CS}}$	log-uniform [0.001–10]	0.004	0.001–0.117	0.156	0.127–0.174
${\text{Nm}}_{\text{SC}}$	log-uniform [0.001–10]	0.340	0.024–2.843	0.515	0.483–0.517

^a^C = Collared, P = Pied, A = Atlas, S = Semicollared, ${\text{N}}_{\text{NOW}}=$ current effective population size (diploid individuals), ${\text{N}}_{\text{ANC}}=$ ancestral effective population size, ${\text{T}}_{\text{SPLIT}}$P/A = split time of pied and Atlas flycatcher (years), ${\text{T}}_{\text{SPLIT}}$PA/C = split time of pied–Atlas and collared flycatcher, ${\text{T}}_{\text{SPLIT}}$PAC/S = split time of pied–Atlas–collared and semicollared flycatcher, ${\text{Nm}}_{\text{XY}}=$ number of migrants per generation in species X from species Y;^b^the prior distributions for the parameter values in the ABC analysis were either uniform or log-uniform within the boundaries provided in squared brackets; ^c^mode of the posterior density distribution from the ABC analysis; ^d^90%-highest posterior density interval from the ABC analysis; ^e^maximum-likelihood parameter estimate from the fastsimcoal2 analysis; ^f^90% confidence interval of maximum-likelihood parameter estimates from 50 repetitions of the subsampling procedure in the fastsimcoal2 analysis.

**Figure 6.**
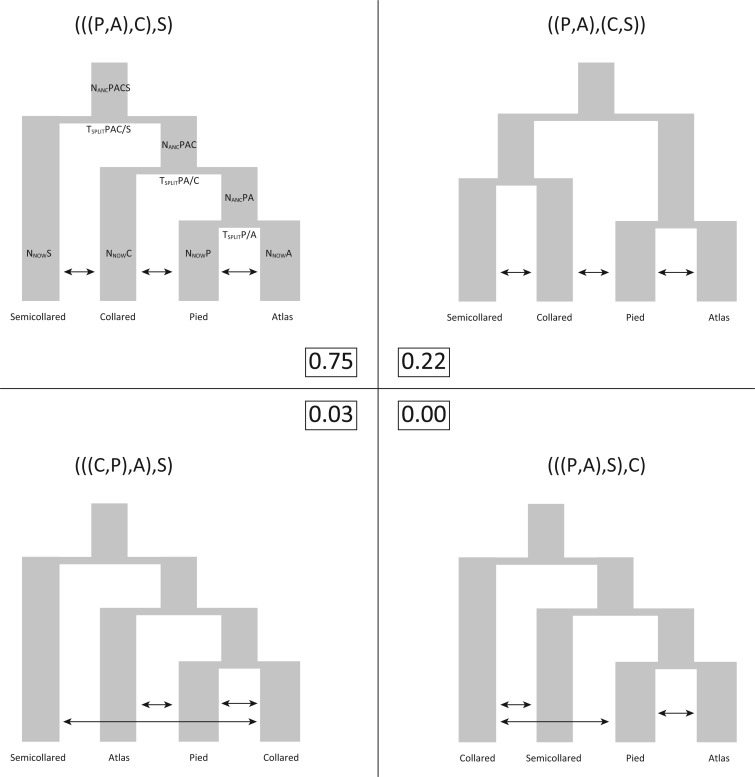
Illustration of the four models used in the ABC model testing. Model parameters are shown for the top left model and correspond to the parameter names in Table 3. The numbers in boxes show the posterior model probabilities from the ABC analysis.

Second, we applied an ABC framework to perform model selection and parameter estimation on demographic models derived from the four most frequent gene tree topologies (Fig. 6). The ABC analysis struggled to distinguish among the four different models, as evidenced by largely overlapping distributions of summary statistics and a misassignment rate of up to 28% in the model selection cross-validation (Figs. S4 and S5). This lack of power likely results from the loss of information when reducing the genomic data to summary statistics. Still, we obtained 75% posterior probability for the same topology as inferred from SNAPP and fastsimcoal2, representing the asymmetric tree with pied and Atlas flycatchers as sister species (Fig. 6). Together with the corresponding symmetric tree (i.e., ((P,A),(C,S))), a species tree with pied and Atlas flycatchers as sister species was supported with 97% posterior probability. Therefore, the by far largest part of the uncertainty in model choice originated from problems differentiating between symmetric and asymmetric species trees with a pied–Atlas pairing, corresponding to the two most frequently observed gene tree topologies based on 10-kb windows (Fig. 3a).

Point estimates of model parameters from the ABC analysis followed a similar pattern as in the fastsimcoal2 analysis, but resulted in about twice lower estimates of current ${\text{N}}_{e}$ in the four species (Table 3). In contrast to the fastsimcoal2 estimate, the internode between the Atlas/pied and (Atlas–pied)/collared splits was characterized by about eight times larger ancestral than current ${\text{N}}_{e}$, resulting in an estimated length of only 0.09 $N_{\text{e}}$ generations. With such a short internode length, very few coalescent events are expected to happen in the ancestral population of pied and Atlas flycatchers, resulting in high frequencies of discordant gene trees due to ILS.

## Discussion

When speciation events occur in quick succession or when established species continue to exchange genes, independent regions along the genome will show phylogenetic relationships that differ from the species tree (Nichols 2001; Degnan and Salter 2005; Degnan and Rosenberg 2009). This stochasticity in lineage sorting between populations and species makes inferences of the sequence of speciation events a challenging task. Despite the emergence of large-scale genomic data sets in many organisms, the problem of species tree reconstruction cannot be easily solved by just considering more data (Degnan and Rosenberg 2009). Rather, under certain topology and branch length combinations, species tree inference from a large number of gene trees can become positively misleading because the most frequently observed gene tree topology does not necessarily correspond to the species tree (Degnan and Rosenberg 2006). Here, capitalizing on a large population genomic data set of four closely related flycatcher species, we were able to show how gene tree topologies vary extensively and at a small physical scale along the genome. Despite the frequent occurrence of every possible tree topology, we reconstructed the underlying species tree with high confidence using a series of coalescent-based approaches taking both ILS and interspecific gene flow into account. Our study thereby illustrates the usefulness of modeling approaches to efficiently extract information about speciation patterns from large genome-wide data sets and provide confident answers even in such closely related and hybridizing systems such as the black-and-white flycatchers.

Widespread incongruence of gene trees from multiple independent loci due to ILS has been recorded in many taxonomic studies (reviewed in Degnan and Rosenberg 2009). This problem is especially pronounced in species with large ${\text{N}}_{e}$, due to the low rate of coalescence in ancestral populations. Furthermore, resolving species relationships in taxa undergoing rapid adaptive radiations has been found to be particularly challenging (e.g., Davies et al. 2004; Ericson et al. 2006; Alfaro et al. 2009). It has been suggested that in such cases the problem of widespread ILS can be alleviated by considering loci in the genome that exhibit reduced $N_{\text{e}}$ as compared with the genome-wide average (Corl and Ellegren 2013; Pease and Hahn 2013; Cruickshank and Hahn 2014). Genome-wide variation of $N_{\text{e}}$ at noncoding sites has been mostly attributed to the effects of selection reducing the diversity of neutral linked sites (Charlesworth et al. 1993; Gossmann et al. 2011; Charlesworth 2012). It follows that sites with low $N_{\text{e}}$ are expected to be found in regions with low recombination rates and/or high density of sites under selection. In such regions, the coalescence rate is increased, causing species to attain reciprocal monophyly faster as compared with regions with higher ${\text{N}}_{e}$. Furthermore, the probability that any particular gene tree yields the same topology as the species tree is increased if $N_{\text{e}}$ was also reduced in the ancestral populations. Such patterns can be expected for background selection, whereby sites under purifying selection reduce the neutral variability at linked sites (Charlesworth et al. 1993).

Our results demonstrate that, contrary to previous suggestions (Corl and Ellegren 2013; Pease and Hahn 2013; Cruickshank and Hahn 2014), regions of reduced ${\text{N}}_{e}$ are not guaranteed to provide a more accurate inference of the species tree. This concerns especially regions that are selected based on extreme values of summary statistics (like nucleotide diversity or ${\text{F}}_{\text{ST}}$), as illustrated by the distribution of gene trees found in differentiation islands. Even though we observe increased species monophyly within differentiation islands, a large variety of tree topologies was recovered. Despite low levels of recombination in these regions, topologies still switch within short physical distances and any of the 15 possible tree topologies can be found. Importantly, restricting the analysis to differentiation islands led to an increase in the most frequently observed gene tree topology, which differed from the top-ranking topology obtained genome-wide. Given that differentiation islands allegedly represent regions of reduced $N_{\text{e}}$, and therefore reduced occurrence of ILS, such a pattern might itself give the impression that the true species tree topology can be recovered with a simple quantification of gene tree topologies from a small number of specifically selected loci. However, as we show here, only explicit modeling of the coalescent process under different species trees can provide a reliable answer regarding the order of speciation events in groups of closely related and genetically connected species.

All four coalescent-based species tree methods resulted in the same species tree topology, supporting pied and Atlas flycatcher as sister taxa. Interestingly, SNAPP, which employs a simple isolation model without gene flow, resulted in the same topology as the models allowing for migration among species. Therefore, in the present case, interspecific gene flow seems to have a negligible impact on the genome-wide distribution of genealogies. This observation is further supported by low estimates of migration rates from the two models with gene flow and in agreement with previous findings pointing to species divergence in isolated glacial refugia (Saetre et al. 2001; Backström et al. 2013; Nadachowska-Brzyska et al. 2013). In this scenario, gene flow among species was limited to sporadic episodes of secondary contact after the northwards expansion during interglacials. Furthermore, in the case of collared and pied flycatchers, hybrids have a reduced fitness as compared with the parental species (Svedin et al. 2008; Wiley et al. 2009), resulting in genome-wide selection against immigrant lineages. Under such an allopatric speciation scenario with both pre- and postzygotic reproductive barriers, low levels of interspecific gene flow can be expected when averaging over the whole genome.

In this study, we focused primarily on ILS and interspecific gene flow as the major processes behind gene tree discordance, which is warranted by the close evolutionary relationships within our study system (Maddison 1997; Degnan and Rosenberg 2009). However, it should be noted that on larger evolutionary time scales, processes related to gene duplication and loss might play a prominent role in shaping gene tree heterogeneity across the genome (Fitch 1970; Goodman et al. 1979). In such cases, approaches that model gene duplication and loss from alignments of gene families might be better suited to account for gene tree discordance in species tree reconstruction (e.g., PHYLDOG; Boussau et al. 2013).

Having obtained a consistent reconstruction of the most likely species tree over multiple modeling approaches, the distribution of gene trees revealed phylogenetic patterns in differentiation islands and on the Z chromosome that go beyond the expectations of a simple reduction in ${\text{N}}_{e}$. Both subsets showed an increase in frequency of the collared–semicollared pairing, resulting in a most frequent gene tree topology different from the inferred species tree. In both cases, the relative increase of the collared–semicollared pairing was accompanied by a reduction in frequency of the collared–pied pairing. Gene trees with a collared–pied pairing are discordant with the inferred species tree, and the relative overrepresentation of this pairing in the whole-genome data set is a strong indicator for gene flow between these two flycatcher species (cf. Backström et al. 2013; Nadachowska-Brzyska et al. 2013). The fact that such signals were diminished on the Z chromosome hints at reduced sex-linked introgression. The Z chromosome in flycatchers has been found to harbor genes involved in pre- and postzygotic reproductive isolation (Saether et al. 2007). Selection against immigrant alleles might therefore be particularly pronounced on the Z chromosome, resulting in a reduced effective gene flow rate (Saetre et al. 2003).

At a first glance, Z-chromosomal loci may seem particularly suitable for species tree reconstruction due to faster lineage sorting and a reduced tendency of introgression compared with autosomal loci. However, several pitfalls are connected to this approach. First, hybrid sterility of the heterogametic sex (females in birds, including flycatchers; Haldane 1922; Svedin et al. 2008) increases the effective rate of gene flow on the Z chromosome relative to autosomes, because the loss of transmission due to sterile female hybrids is relatively less for Z chromosomes than autosomes. Such a pattern might explain the increased occurrence of collared–semicollared pairings in Z-chromosomal gene trees to the point that the symmetric ((P,A),(C,S)) topology becomes the most frequent. Alternatively, this pattern might be explained by positive selection of the same alleles in collared and semicollared flycatchers. Due to the relatively recent split times and large ancestral population sizes, many positively selected alleles in species-specific sweeps are likely to originate from standing genetic variation in the common ancestor. The nonrandom sorting of such ancestral variation according to similarities in ecology or life history could therefore explain localized patterns of reduced genetic divergence conflicting with the species tree. This particularly concerns regions showing signatures of selection, such as reduced nucleotide diversity found in differentiation islands in flycatchers. A similar pattern was found in African cichlid fish (Brawand et al. 2014), where regions of elevated differentiation between rapidly radiating species contained phylogenetic signals strongly discordant with the taxonomic classification.

Our study clearly demonstrates the power of coalescent-based modeling to resolve species trees even in closely related and hybridizing species, provided that data from a sufficient number of independent and neutrally evolving loci is available. By re-subsampling our whole-genome data set, we showed that a few hundred independent sequence loci are sufficient to confidently infer the species tree in species with low rates of interspecific gene flow. Although employing loci with reduced recombination rate and ${\text{N}}_{e}$ can in theory alleviate the problem of ILS, such loci are potentially under the influence of selection, which might lead to strong biases in the reconstruction of species relationships. Particular caution is warranted when applying concatenation (supermatrix) approaches to such data, because in our case concatenation was more prone to yield erroneous species relationships in low-recombination regions than coalescent-based methods. Thus, for reliable species tree inference, we recommend that data on the order of a few hundred randomly selected and genome-wide distributed loci are obtained. An increasing range of coalescent-based modeling methods can easily deal with such a number of loci sequenced in multiple individuals per species.

Despite recent advances, a major bottleneck to the widespread application of coalescent-based models for species tree reconstruction remains, as computational constraints limit current implementations of complex models to a small number of species. Furthermore, even models that take both ILS and interspecific gene flow into account are simple representations of the demographic history of natural populations. For instance, speciation might involve complex demographic processes, such as series of bottlenecks and subsequent population expansions associated with founder events (Mayr 1942), a slow decay of genetic exchange in speciation-with-gene-flow scenarios (Feder et al. 2012), or genetic exchange upon secondary contact after a period of geographic isolation. Deviations from the relatively simple models assumed in most model-based approaches might negatively influence species tree inference. Additionally, these factors are not just nuisance parameters but themselves provide crucial information about the evolutionary forces involved in the speciation process. In the future, complex yet computationally efficient species tree models in combination with extensive population genomic data sets might allow us to obtain a much more refined picture of the evolutionary history in a wide range of species.

## Supplementary Material

Supplementary material, including data files and online-only appendices can be found in the Dryad Data Repository: http://dx.doi.org/10.5061/dryad.b6gj8. Raw read data and alignment files have been deposited in the Sequence Read Archive (http://www.ebi.ac.uk/ena) under the study accession ID PRJEB7359.

## Funding

This work was supported by an Advanced Investigator Grant (NEXTGENMOLECOL) from the European Research Council, a Wallenberg Scholar Award from the Knut and Alice Wallenberg Foundation and from the Swedish Research Council (2007-8731, 2010-5650 and 2013-8271). R.B. was supported by the Swiss National Science Foundation, grants (PBLAP3-134299) and (PBLAP3-140171).

## References

[B1] Alfaro M.E. Santini F. Brock C. Alamillo H. Dornburg A. Rabosky D.L. Carnevale G. Harmon L.J. 2009 Nine exceptional radiations plus high turnover explain species diversity in jawed vertebrates. Proc. Natl. Acad. Sci. USA 106:13410–13414. 19633192 10.1073/pnas.0811087106 PMC2715324

[B2] Avise J.C. Shapira J.F. Daniel S.W. Aquadro C.F. Lansman R.A. 1983 Mitochondrial DNA differentiation during the speciation process in Peromyscus. Mol. Biol. Evol. 1:38–56. 6400647 10.1093/oxfordjournals.molbev.a040301

[B3] Backström N. Lindell J. Zhang Y. Palkopoulou E. Qvarnström A. Saetre G.P. Ellegren H. 2010 A high-density scan of the Z chromosome in Ficedula Flycatchers reveals candidate loci for diversifying selection. Evolution 64:3461–3475. 20629730 10.1111/j.1558-5646.2010.01082.x

[B4] Backström N. Saetre G.P. Ellegren H. 2013 Inferring the demographic history of European Ficedula flycatcher populations. BMC Evol. Biol. 13:2 doi:10.1186/1471-2148-13-2. 23282063 10.1186/1471-2148-13-2 PMC3556140

[B5] Beaumont M.A. Zhang W.Y. Balding D.J. 2002 Approximate Bayesian computation in population genetics. Genetics 162:2025–2035. 12524368 10.1093/genetics/162.4.2025 PMC1462356

[B6] Borge T. Lindroos K. Nadvornik P. Syvanen A.C. Saetre G.P. 2005 Amount of introgression in flycatcher hybrid zones reflects regional differences in pre and post-zygotic barriers to gene exchange. J. Evol. Biol. 18:1416–1424. 16313454 10.1111/j.1420-9101.2005.00964.x

[B7] Bouckaert R.R. 2010 DensiTree: making sense of sets of phylogenetic trees. Bioinformatics 26:1372–1373. 20228129 10.1093/bioinformatics/btq110

[B8] Boulesteix A.L. Strimmer K. 2007 Partial least squares: a versatile tool for the analysis of high-dimensional genomic data. Brief. Bioinformatics 8:32–44. 16772269 10.1093/bib/bbl016

[B9] Boussau B. Szollosi G.J. Duret L. Gouy M. Tannier E. Daubin V. 2013 Genome-scale coestimation of species and gene trees. Genome Res. 23:323–330. 23132911 10.1101/gr.141978.112 PMC3561873

[B10] Brawand D. Wagner C.E. Li Y.I. Malinsky M. Keller I. Fan S. Simakov O. Ng A.Y. Lim Z.W. Bezault E. Turner-Maier J. Johnson J. Alcazar R. Noh H.J. Russell P. Aken B. Alfoldi J. Amemiya C. Azzouzi N. Baroiller J.F. Barloy-Hubler F. Berlin A. Bloomquist R. Carleton K.L. Conte M.A. D'Cotta H. Eshel O. Gaffney L. Galibert F. Gante H.F. Gnerre S. Greuter L. Guyon R. Haddad N.S. Haerty W. Harris R.M. Hofmann H.A. Hourlier T. Hulata G. Jaffe D.B. Lara M. Lee A.P., MacCallum I. Mwaiko S. Nikaido M. Nishihara H. Ozouf-Costaz C. Penman D.J. Przybylski D. Rakotomanga M. Renn S.C. Ribeiro F.J. Ron M. Salzburger W. Sanchez-Pulido L. Santos M.E. Searle S. Sharpe T. Swofford R. Tan F.J. Williams L. Young S. Yin S. Okada N. Kocher T.D. Miska E.A. Lander E.S. Venkatesh B. Fernald R.D. Meyer A. Ponting C.P. Streelman J.T. Lindblad-Toh K. Seehausen O. Di Palma F. 2014 The genomic substrate for adaptive radiation in African cichlid fish. Nature, 513:375–381. 25186727 10.1038/nature13726 PMC4353498

[B11] Bryant D. Bouckaert R. Felsenstein J. Rosenberg N.A. RoyChoudhury A. 2012 Inferring species trees directly from biallelic genetic markers: bypassing gene trees in a full coalescent analysis. Mol. Biol. Evol. 29:1917–1932. 22422763 10.1093/molbev/mss086 PMC3408069

[B12] Charlesworth B. 2012 The effects of deleterious mutations on evolution at linked sites. Genetics 190:5–22. 22219506 10.1534/genetics.111.134288 PMC3249359

[B13] Charlesworth B. Morgan M.T. Charlesworth D. 1993 The effect of deleterious mutations on neutral molecular variation. Genetics 134:1289–1303. 8375663 10.1093/genetics/134.4.1289 PMC1205596

[B14] Chen H. Boutros P.C. 2011 VennDiagram: a package for the generation of highly-customizable Venn and Euler diagrams in R. BMC Bioinformatics 12:35 doi:10.1186/1471-2105-12-35. 21269502 10.1186/1471-2105-12-35 PMC3041657

[B15] Corl A. Ellegren H. 2013 Sampling strategies for species trees: the effects on phylogenetic inference of the number of genes, number of individuals, and whether loci are mitochondrial, sex-linked, or autosomal. Mol. Phylogenet. Evol. 67:358–366. 23410742 10.1016/j.ympev.2013.02.002

[B16] Cruickshank T.E. Hahn M.W. 2014 Reanalysis suggests that genomic islands of speciation are due to reduced diversity, not reduced gene flow. Mol. Ecol. 23:3133–3157. 24845075 10.1111/mec.12796

[B17] Csillery K. Francois O. Blum M.G.B. 2012 abc: an R package for approximate Bayesian computation (ABC). Methods Ecol. Evol. 3:475–479. 10.1016/j.tree.2010.04.001 20488578

[B18] Cummings M.P. Neel M.C. Shaw K.L. 2008 A genealogical approach to quantifying lineage divergence. Evolution 62:2411–2422. 18564377 10.1111/j.1558-5646.2008.00442.x

[B19] Davies T.J. Barraclough T.G. Chase M.W. Soltis P.S. Soltis D.E. Savolainen V. 2004 Darwin's abominable mystery: insights from a supertree of the angiosperms. Proc. Natl. Acad. Sci. USA 101:1904–1909. 14766971 10.1073/pnas.0308127100 PMC357025

[B20] Degnan J.H. Rosenberg N.A. 2006 Discordance of species trees with their most likely gene trees. PLoS Genet. 2:762–768. 10.1371/journal.pgen.0020068 PMC1464820 16733550

[B21] Degnan J.H. Rosenberg N.A. 2009 Gene tree discordance, phylogenetic inference and the multispecies coalescent. Trends Ecol. Evol. 24:332–340. 19307040 10.1016/j.tree.2009.01.009

[B22] Degnan J.H. Salter L.A. 2005 Gene tree distributions under the coalescent process. Evolution 59:24–37. 15792224

[B23] Edwards S.V. 2009 Is a new and general theory of molecular systematics emerging? Evolution 63:1–19. 19146594 10.1111/j.1558-5646.2008.00549.x

[B24] Edwards S.V. Liu L. Pearl D.K. 2007 High-resolution species trees without concatenation. Proc. Natl. Acad. Sci. USA 104:5936–5941. 17392434 10.1073/pnas.0607004104 PMC1851595

[B25] Ellegren H. Gustafsson L. Sheldon B.C. 1996 Sex ratio adjustment in relation to paternal attractiveness in a wild bird population. Proc. Natl. Acad. Sci. USA 93:11723–11728. 8876204 10.1073/pnas.93.21.11723 PMC38125

[B26] Ellegren H. Smeds L. Burri R. Olason P.I. Backstrom N. Kawakami T. Kunstner A. Makinen H. Nadachowska-Brzyska K. Qvarnstrom A. Uebbing S. Wolf J.B.W. 2012 The genomic landscape of species divergence in Ficedula flycatchers. Nature 491:756–760. 23103876 10.1038/nature11584

[B27] Ericson P.G.P. Anderson C.L. Britton T. Elzanowski A. Johansson U.S. Kallersjo M. Ohlson J.I. Parsons T.J. Zuccon D. Mayr G. 2006 Diversification of Neoaves: integration of molecular sequence data and fossils. Biol. Lett. 2:543–U541. 17148284 10.1098/rsbl.2006.0523 PMC1834003

[B28] Excoffier L. Dupanloup I. Huerta-Sanchez E. Sousa V.C. Foll M. 2013 Robust demographic inference from genomic and SNP Data. PLoS Genet 9(10):e1003905 doi:10.1371/journal.pgen.1003905. 24204310 10.1371/journal.pgen.1003905 PMC3812088

[B29] Excoffier L. Smouse P.E. Quattro J.M. 1992 Analysis of molecular variance inferred from metric distances among DNA haplotypes - application to human mitochondrial DNA restriction data. Genetics 131:479–491. 1644282 10.1093/genetics/131.2.479 PMC1205020

[B30] Feder J.L. Egan S.P. Nosil P. 2012 The genomics of speciation-with-gene-flow. Trends Genet. 28:342–350. 22520730 10.1016/j.tig.2012.03.009

[B31] Felsenstein J. 1981 Evolutionary trees from DNA sequences: a maximum-likelihood approach. J. Mol. Evol. 17:368–376. 7288891 10.1007/BF01734359

[B32] Felsenstein J. 2004 Inferring phylogenies. Sunderland, MA: Sinauer Associates.

[B33] Ferris S.D. Wilson A.C. Brown W.M. 1981 Evolutionary tree for apes and humans based on cleavage maps of mitochondrial-DNA. Proc. Natl. Acad. Sci. USA 78:2432–2436. 6264476 10.1073/pnas.78.4.2432 PMC319360

[B34] Fitch W.M. 1970 Distinguishing homologous from analogous proteins. Syst. Biol. 19:99–113. 5449325

[B35] Gatesy J. Springer M.S. 2014 Phylogenetic analysis at deep timescales: unreliable gene trees, bypassed hidden support, and the coalescence/concatalescence conundrum. Mol. Phylogenet. Evol. 80:231–266. 25152276 10.1016/j.ympev.2014.08.013

[B36] Golding B. Felsenstein J. 1990 A maximum-likelihood approach to the detection of selection from a phylogeny. J. Mol. Evol. 31:511–523. 2176699 10.1007/BF02102078

[B37] Goodman M. Czelusniak J. Moore G.W. Romeroherrera A.E. Matsuda G. 1979 Fitting the gene lineage into its species lineage, a parsimony strategy illustrated by cladograms constructed from globin sequences. Syst. Zool. 28:132–163.

[B38] Gossmann T.I. Woolfit M. Eyre-Walker A. 2011 Quantifying the variation in the effective population size within a genome. Genetics 189:1389–1402. 21954163 10.1534/genetics.111.132654 PMC3241429

[B39] Grant P.R. Grant B.R. 1992 Hybridization of bird species. Science 256:193–197. 17744718 10.1126/science.256.5054.193

[B40] Griffiths R.C. Marjoram P. 1996 Ancestral inference from samples of DNA sequences with recombination. J. Comput. Biol. 3:479–502. 9018600 10.1089/cmb.1996.3.479

[B41] Haldane J.B.S. 1922 Sex ratio and unisexual sterility in hybrid animals. J. Genet. 12:101–109.

[B42] Heled J. Drummond A.J. 2010 Bayesian inference of species trees from multilocus data. Mol. Biol. Evol. 27:570–580. 19906793 10.1093/molbev/msp274 PMC2822290

[B43] Hey J. 2006 Recent advances in assessing gene flow between diverging populations and species. Curr. Opin. Genet. Dev. 16:592–596. 17055250 10.1016/j.gde.2006.10.005

[B44] Hey J. Nielsen R. 2004 Multilocus methods for estimating population sizes, migration rates and divergence time, with applications to the divergence of *Drosophila pseudoobscura* and *D. persimilis*. Genetics 167:747–760. 15238526 10.1534/genetics.103.024182 PMC1470901

[B45] Hogner S. Saether S.A. Borge T. Bruvik T. Johnsen A. Saetre G.P. 2012 Increased divergence but reduced variation on the Z chromosome relative to autosomes in Ficedula flycatchers: differential introgression or the faster-Z effect? Ecol. Evol. 2:379–396. 22423331 10.1002/ece3.92 PMC3298950

[B46] Hudson R.R. Futuyma D.J. Antonovics J.D. 1990 Gene genealogies and the coalescent process. Oxford surveys in evolutionary biology. New York: Oxford University Press p. 1–44.

[B47] Hudson R.R. 2002 Generating samples under a Wright-Fisher neutral model of genetic variation. Bioinformatics 18:337–338. 11847089 10.1093/bioinformatics/18.2.337

[B48] Johnson J.B. Omland K.S. 2004 Model selection in ecology and evolution. Trends Ecol. Evol. 19:101–108. 16701236 10.1016/j.tree.2003.10.013

[B49] Junier T. Zdobnov E.M. 2010 The Newick utilities: high-throughput phylogenetic tree processing in the Unix shell. Bioinformatics 26:1669–1670. 20472542 10.1093/bioinformatics/btq243 PMC2887050

[B50] Kaplan N.L. Hudson R.R. Langley C.H. 1989 The “hitchhiking effect” revisited. Genetics 123:887–899. 2612899 10.1093/genetics/123.4.887 PMC1203897

[B51] Kawakami T. Backstrom N. Burri R. Husby A. Olason P. Rice A.M. Alund M. Qvarnstrom A. Ellegren H. 2014a Estimation of linkage disequilibrium and interspecific gene flow in *Ficedula *flycatchers by a newly developed 50k single-nucleotide polymorphism array. Mol. Ecol. Resour. 14:1248–1260. 24784959 10.1111/1755-0998.12270 PMC4368375

[B52] Kawakami T. Smeds L. Backstrom N. Husby A. Qvarnstrom A. Mugal C.F. Olason P. Ellegren H. 2014b A high-density linkage map enables a second-generation collared flycatcher genome assembly and reveals the patterns of avian recombination rate variation and chromosomal evolution. Mol. Ecol. 23:4035–4058. 24863701 10.1111/mec.12810 PMC4149781

[B53] Kubatko L.S. Degnan J.H. 2007 Inconsistency of phylogenetic estimates from concatenated data under coalescence. Syst. Biol. 56:17–24. 17366134 10.1080/10635150601146041

[B54] Leache A.D. Rannala B. 2011 The accuracy of species tree estimation under simulation: a comparison of methods. Syst. Biol. 60:126–137. 21088009 10.1093/sysbio/syq073

[B55] Leuenberger C. Wegmann D. 2010 Bayesian computation and model selection without likelihoods. Genetics 184:243–252. 19786619 10.1534/genetics.109.109058 PMC2815920

[B56] Li H. Durbin R. 2009 Fast and accurate short read alignment with Burrows-Wheeler transform. Bioinformatics 25:1754–1760. 19451168 10.1093/bioinformatics/btp324 PMC2705234

[B57] Liu L. 2008 BEST: Bayesian estimation of species trees under the coalescent model. Bioinformatics 24:2542–2543. 18799483 10.1093/bioinformatics/btn484

[B58] Liu L. Pearl D.K. 2007 Species trees from gene trees: reconstructing Bayesian posterior distributions of a species phylogeny using estimated gene tree distributions. Syst. Biol. 56:504–514. 17562474 10.1080/10635150701429982

[B59] Liu L. Yu L.L. Pearl D.K. Edwards S.V. 2009 Estimating species phylogenies using coalescence times among sequences. Syst. Biol. 58:468–477. 20525601 10.1093/sysbio/syp031

[B60] Liu L.A. Yu L.L. Edwards S.V. 2010 A maximum pseudo-likelihood approach for estimating species trees under the coalescent model. BMC Evol. Biol. 10:302 doi:10.1186/1471-2148-10-302. 20937096 10.1186/1471-2148-10-302 PMC2976751

[B61] Lundberg A. Alatalo R.V. 1992 The pied flycatcher. London, UK: T & AD Poyser.

[B62] Maddison W.P. 1997 Gene trees in species trees. Syst. Biol. 46:523–536.

[B63] Marjoram P. Molitor J. Plagnol V. Tavare S. 2003 Markov chain Monte Carlo without likelihoods. Proc. Natl. Acad. Sci. USA 100:15324–15328. 14663152 10.1073/pnas.0306899100 PMC307566

[B64] Mayr E. 1942 Systematics and the origin of species from the viewpoint of a zoologist. New York: Columbia University Press.

[B65] McKenna A. Hanna M. Banks E. Sivachenko A. Cibulskis K. Kernytsky A. Garimella K. Altshuler D. Gabriel S. Daly M. DePristo M.A. 2010 The Genome Analysis Toolkit: A MapReduce framework for analyzing next-generation DNA sequencing data. Genome Research, 20:1297–1303. 20644199 10.1101/gr.107524.110 PMC2928508

[B66] Mevik B.H. Wehrens R. 2007 The pls package: principal component and partial least squares regression in R. J. Stat. Software 18:1–24.

[B67] Nachman M.W. Payseur B.A. 2012 Recombination rate variation and speciation: theoretical predictions and empirical results from rabbits and mice. Philos. Trans. R. Soc. B Biol. Sci. 367:409–421. 10.1098/rstb.2011.0249 PMC3233716 22201170

[B68] Nadachowska-Brzyska K. Burri R. Olason P.I. Kawakami T. Smeds L. Ellegren H. 2013 Demographic divergence history of pied flycatcher and collared flycatcher inferred from whole-genome re-sequencing data. PLoS Genet. 9(11):e1003942 doi:10.1371/journal.pgen.1003942. 24244198 10.1371/journal.pgen.1003942 PMC3820794

[B69] Nichols R. 2001 Gene trees and species trees are not the same. Trends Ecol. Evol. 16:358–364. 11403868 10.1016/s0169-5347(01)02203-0

[B70] Nosil P. Funk D.J. Ortiz-Barrientos D. 2009 Divergent selection and heterogeneous genomic divergence. Mol. Ecol. 18:375–402. 19143936 10.1111/j.1365-294X.2008.03946.x

[B71] Pamilo P. Nei M. 1988 Relationships between gene trees and species trees. Mol. Biol. Evol. 5:568–583. 3193878 10.1093/oxfordjournals.molbev.a040517

[B72] Pease J.B. Hahn M.W. 2013 More accurate phylogenies inferred from low-recombination regions in the presence of incomplete lineage sorting. Evolution 67:2376–2384. 23888858 10.1111/evo.12118 PMC3929462

[B73] Qvarnstrom A. Part T. Sheldon B.C. 2000 Adaptive plasticity in mate preference linked to differences in reproductive effort. Nature 405:344–347. 10830962 10.1038/35012605

[B74] Qvarnstrom A. Rice A.M. Ellegren H. 2010 Speciation in *Ficedula *flycatchers. Philos. Trans. R Soc. Lond. B Biol. Sci. 365:1841–1852. 20439285 10.1098/rstb.2009.0306 PMC2871891

[B75] R Development Core Team 2010 R: a language and environment for statistical computing. Vienna, Austria: R Foundation for Statistical Computing.

[B76] Rambaut A., Drummond A.J. 2007. Tracer v1.4. Available from: http://beast.bio.ed.ac.uk/Tracer.

[B77] Rannala B. Yang Z.H. 2003 Bayes estimation of species divergence times and ancestral population sizes using DNA sequences from multiple loci. Genetics 164:1645–1656. 12930768 10.1093/genetics/164.4.1645 PMC1462670

[B78] Rannala B. Yang Z.H. 2008 Phylogenetic inference using whole Genomes. Annu. Rev. Genomics Hum. Genet. 9:217–231. 18767964 10.1146/annurev.genom.9.081307.164407

[B79] Robinson J.D. Bunnefeld L. Hearn J. Stone G.N. Hickerson M.J. 2014 ABC inference of multi-population divergence with admixture from unphased population genomic data. Mol. Ecol. 23:4458–4471. 25113024 10.1111/mec.12881 PMC4285295

[B80] Saether S.A. Saetre G.P. Borge T. Wiley C. Svedin N. Andersson G. Veen T. Haavie J. Servedio M.R. Bures S. Kral M. Hjernquist M.B. Gustafsson L. Traff J. Qvarnstrom A. 2007 Sex chromosome-linked species recognition and evolution of reproductive isolation in flycatchers. Science, 318:95–97. 17916732 10.1126/science.1141506

[B81] Saetre G.P. Borge T. Lindell J. Moum T. Primmer C.R. Sheldon B.C. Haavie J. Johnsen A. Ellegren H. 2001 Speciation, introgressive hybridization and nonlinear rate of molecular evolution in flycatchers. Mol. Ecol. 10:737–749. 11298984 10.1046/j.1365-294x.2001.01208.x

[B82] Saetre G.P. Borge T. Lindroos K. Haavie J. Sheldon B.C. Primmer C. Syvanen A.C. 2003 Sex chromosome evolution and speciation in Ficedula flycatchers. Proc. R Soc. London B Biol. Sci. 270:53–59. 10.1098/rspb.2002.2204 PMC1691206 12590771

[B83] Saetre G.P. Moum T. Bures S. Kral M. Adamjan M. Moreno J. 1997 A sexually selected character displacement in flycatchers reinforces premating isolation. Nature 387:589–592.

[B84] Saetre G.P. Saether S.A. 2010 Ecology and genetics of speciation in *Ficedula* flycatchers. Mol. Ecol. 19:1091–1106. 20163542 10.1111/j.1365-294X.2010.04568.x

[B85] Scally A. Dutheil J.Y. Hillier L.W. Jordan G.E. Goodhead I. Herrero J. Hobolth A. Lappalainen T. Mailund T. Marques-Bonet T. McCarthy S. Montgomery S.H. Schwalie P.C. Tang Y.A. Ward M.C. Xue Y.L. Yngvadottir B. Alkan C. Andersen L.N. Ayub Q. Ball E.V. Beal K. Bradley B.J. Chen Y. Clee C.M. Fitzgerald S. Graves T.A. Gu Y. Heath P. Heger A. Karakoc E. Kolb-Kokocinski A. Laird G.K. Lunter G. Meader S. Mort M. Mullikin J.C. Munch K. O'Connor T.D. Phillips A.D. Prado-Martinez J. Rogers A.S. Sajjadian S. Schmidt D. Shaw K. Simpson J.T. Stenson P.D. Turner D.J. Vigilant L. Vilella A.J. Whitener W. Zhu B.L. Cooper D.N. de Jong P. Dermitzakis E.T. Eichler E.E. Flicek P. Goldman N. Mundy N.I. Ning Z.M. Odom D.T. Ponting C.P. Quail M.A. Ryder O.A. Searle S.M. Warren W.C. Wilson R.K. Schierup M.H. Rogers J. Tyler-Smith C. Durbin R. 2012 Insights into hominid evolution from the gorilla genome sequence. Nature, 483:169–175. 22398555 10.1038/nature10842 PMC3303130

[B86] Scheet P. Stephens M. 2006 A fast and flexible statistical model for large-scale population genotype data: applications to inferring missing genotypes and haplotypic phase. Am. J. Hum. Genet. 78:629–644. 16532393 10.1086/502802 PMC1424677

[B87] Smeds L. Warmuth V. Bolivar P. Uebbing S. Burri R. Suh A. Nater A. Bures S. Garamszegi L.Z. Hogner S. Moreno J. Qvarnstrom A. Ruzic M. Saether S.A. Saetre G.P. Torok J. Ellegren H. 2015 Evolutionary analysis of the female-specific avian W chromosome. Nat Commun, 6:7330. 26040272 10.1038/ncomms8330 PMC4468903

[B88] Stajich J.E. Block D. Boulez K. Brenner S.E. Chervitz S.A. Dagdigian C. Fuellen G. Gilbert J.G.R. Korf I. Lapp H. Lehvaslaiho H. Matsalla C. Mungall C.J. Osborne B.I. Pocock M.R. Schattner P. Senger M. Stein L.D. Stupka E. Wilkinson M.D. Birney E. 2002 The bioperl toolkit: Perl modules for the life sciences. Genome Research, 12:1611–1618. 12368254 10.1101/gr.361602 PMC187536

[B89] Stamatakis A. 2014 RAxML version 8: a tool for phylogenetic analysis and post-analysis of large phylogenies. Bioinformatics 30:1312–1313. 24451623 10.1093/bioinformatics/btu033 PMC3998144

[B90] Svedin N. Wiley C. Veen T. Gustafsson L. Qvarnstrom A. 2008 Natural and sexual selection against hybrid flycatchers. Proc. R. Soc. Lond. B Biol. Sci. 275:735–744. 10.1098/rspb.2007.0967 PMC2596841 18211878

[B91] Tajima F. 1983 Evolutionary relationship of DNA-sequences in finite populations. Genetics 105:437–460. 6628982 10.1093/genetics/105.2.437 PMC1202167

[B92] Veen T. Borge T. Griffith S.C. Saetre G.P. Bures S. Gustafsson L. Sheldon B.C. 2001 Hybridization and adaptive mate choice in flycatchers. Nature 411:45–50. 11333971 10.1038/35075000

[B93] Via S. West J. 2008 The genetic mosaic suggests a new role for hitchhiking in ecological speciation. Mol. Ecol. 17:4334–4345. 18986504 10.1111/j.1365-294X.2008.03921.x

[B94] Wegmann D. Leuenberger C. Excoffier L. 2009 Efficient approximate Bayesian computation coupled with Markov chain Monte Carlo without likelihood. Genetics 182:1207–1218. 19506307 10.1534/genetics.109.102509 PMC2728860

[B95] Wiley C. Qvarnstrom A. Andersson G. Borge T. Saetre G.P. 2009 Postzygotic isolation over multiple generations of hybrid descendents in a natural hybrid zone: how well do single-generation estimates reflect reproductive isolation? Evolution 63:1731–1739. 19245675 10.1111/j.1558-5646.2009.00674.x

[B96] Wu C.I. Ting C.T. 2004 Genes and speciation. Nat. Rev. Genet. 5:114–122. 14735122 10.1038/nrg1269

[B97] Yu Y. Cuong T. Degnan J.H. Nakhleh L. 2011 Coalescent histories on phylogenetic networks and detection of hybridization despite incomplete lineage sorting. Syst. Biol. 60:138–149. 21248369 10.1093/sysbio/syq084 PMC3167682

[B98] Zheng C.Z. Kuhner M.K. Thompson E.A. 2014 Bayesian inference of local trees along chromosomes by the sequential Markov coalescent. J. Mol. Evol. 78:279–292. 24817610 10.1007/s00239-014-9620-5 PMC4104301

